# Regulation of Ribosomal Protein Synthesis in Mycobacteria: The Autogenous Control of *rpsO*

**DOI:** 10.3390/ijms22189679

**Published:** 2021-09-07

**Authors:** Leonid V. Aseev, Ludmila S. Koledinskaya, Oksana S. Bychenko, Irina V. Boni

**Affiliations:** Shemyakin-Ovchinnikov Institute of Bioorganic Chemistry RAS, 117997 Moscow, Russia; leroymail@gmail.com (L.V.A.); TchufistovaLS@yandex.ru (L.S.K.); bychenko.oksana@gmail.com (O.S.B.)

**Keywords:** mycobacteria, ribosomal proteins, autogenous regulation, shuttle vectors

## Abstract

The autogenous regulation of ribosomal protein (r-protein) synthesis plays a key role in maintaining the stoichiometry of ribosomal components in bacteria. In this work, taking the *rpsO* gene as a classic example, we addressed for the first time the in vivo regulation of r-protein synthesis in the mycobacteria *M. smegmatis* (*Msm*) and *M. tuberculosis* (*Mtb*). We used a strategy based on chromosomally integrated reporters under the control of the *rpsO* regulatory regions and the ectopic expression of *Msm* S15 to measure its impact on the reporter expression. Because the use of *E.* *coli* as a host appeared inefficient, a fluorescent reporter system was developed by inserting *Msm* or *Mtb* *rpsO-egfp* fusions into the *Msm* chromosome and expressing *Msm* S15 or *E. coli* S15 in trans from a novel replicative shuttle vector, pAMYC. The results of the eGFP expression measurements in *Msm* cells provided evidence that the *rpsO* gene in *Msm* and *Mtb* was feedback-regulated at the translation level. The mutagenic analysis showed that the folding of *Msm* *rpsO* 5′UTR in a pseudoknot appeared crucial for repression by both *Msm* S15 and *E. coli* S15, thus indicating a striking resemblance of the *rpsO* feedback control in mycobacteria and in *E. coli*.

## 1. Introduction

Bacterial ribosomes have been the targets of a majority of reported clinical antibiotics to date; hence, ribosome biogenesis and its regulation are central to the development of new antimicrobials. The biogenesis of ribosomes in bacteria is energetically costly as it requires the balanced synthesis of three rRNA molecules and multiple ribosomal proteins (r-proteins) in stoichiometric amounts. Among the mechanisms maintaining the coordinated synthesis of ribosomal components, the role of the autogenous control of r-protein operons is widely recognized [1,2,3,4]. The ability of r-proteins synthesized in excess over rRNA to inhibit the expression of their own mRNAs has already been shown for most *E. coli* r-protein operons [2,3,5,6,7,8]. However, our knowledge of the r-protein-mediated regulation is based mainly on investigations conducted on *E. coli* and its close relatives in the γ-proteobacteria, or, to a lesser extent, on *Bacilli*, low-GC Gram-positive organisms. Almost no information is available for other bacterial phyla, including Actinobacteria, which are Gram-positive organisms with a high GC content. This phylum comprises many human pathogens, e.g., *Mycobacterium tuberculosis* and *Mycobacterium leprae* in the genus *Mycobacterium*, which highlights the importance of studies on the mechanisms of mycobacterial gene expression and its regulation.

One of the most thoroughly studied cases in the autogenous regulation of bacterial r-proteins is the *rpsO* gene-encoding r-protein uS15, a primary r-protein in the assembly of the 30S ribosomal subunit. The details of the uS15-mediated autogenous control have been examined in numerous works dedicated to *rpsO* expression regulation in *E. coli* [9,10,11,12,13,14], *Bacillus stearothermophilus* [15,16], *Geobacillus kaustophilus* [17], *Thermus thermophilus* [18], and *Rhizobium radiobacter* [19]. The *rpsO* regulation in all these cases operates at the translation initiation level through the binding of uS15 to specific regulatory structures in the 5′ untranslated region (5′UTR) of the *rpsO* mRNA, leading to the inhibition of translation, either by the ribosome “entrapment” in a non-productive complex (*E. coli*, see [9,13,14]) or by direct competition with the ribosome binding (*Th. thermophilus*, see [18]).

During the 30S ribosome assembly, uS15 binds to a highly evolutionary, conserved central domain of the 16S rRNA. The interaction of uS15 with its rRNA target is well documented: The major contribution to binding is provided by the highly conserved three-helix junction (h20, h21, h22); this interaction is required for the subsequent binding of other proteins (e.g., bS6, bS18) necessary for the formation of the 30S subunit platform. A relatively modest input is provided by the uS15 recognition of a universally conserved U-G/C-G motif in h22 ([12] and references therein). Unlike the high conservation level of the 16S rRNA targets for uS15, the regulatory structures within the 5′UTRs of the *rpsO* mRNAs widely vary both at the primary and secondary structure levels, suggesting a high S15-RNA interaction plasticity [19,20]. Thus, in *E. coli*, the extent of the similarity between the uS15 binding sites on 16S rRNA and on its own mRNA is not high—the only signal common between the two targets is a U-G/C-G motif that contributes modestly to rRNA binding but is crucial for mRNA recognition. The *E. coli* regulatory site (operator) for S15 is a pseudoknot which is stabilized upon uS15 binding, thus preventing the formation of the active initiation complex [9,10,11,12,13,14]. In contrast, the operator structures for uS15 on the *B. stearothermophilus* and *Th. thermophilus rpsO* mRNAs are organized in three-way junction motifs, similar in the secondary (but not primary) structure to the respective 16S rRNA binding regions for uS15 [15,16,18]. The stabilization of the three-helix junction on the mRNA by uS15 binding may prevent the ribosome binding to initiate translation [18]. It is important to enlarge the list of regulatory structures on the natural *rpsO* mRNAs from other bacterial phyla in order to find the common signals providing autogenous regulation by uS15. Recently, a computational analysis of the *rpsO* 5′UTRs predicted the presence of the conserved structural elements in Actinobacteria, indicating a high probability for *rpsO* autogenous regulation in this phylum; however, this has not yet been confirmed experimentally [19].

In this article, taking the *rpsO* gene as a classic example, we address the in vivo regulation of r-protein synthesis in *M. smegmatis* (*Msm*) and *M. tuberculosis* (*Mtb*). We used a previously developed strategy based on chromosomally integrated reporter genes under the control of the *rpsO* regulatory regions and the ectopic expression of *Msm* S15 to measure its effect on reporter expression. This approach allows for a quantitative evaluation of the impact of the excess r-protein on the efficiency of its own mRNA regulatory region. By using *E. coli* as a host, we demonstrated an inhibiting effect of *Msm* S15 *in trans* on the *Msm rpsO’-‘lacZ* expression. However, expression of the *Msm rpsO-lacZ* reporter in *E. coli* turned out to be inefficient, necessitating the development of a cognate system based on *Msm* as an efficient host for mycobacterial gene expression [21]. We developed the fluorescence reporter assay by modifying the integrative shuttle vector pMV306 [22] in order to transfer the reporters *Msm* (or *Mtb*) *rpsO-egfp* onto the *Msm* chromosome; to provide the expression of *Msm* S15 *in trans*, we created a new *E. coli*-mycobacteria replicative shuttle vector, the pAMYC. The results of fluorescence measurements demonstrated that *Msm* and *Mtb rpsO* genes are negatively regulated by both *Msm* S15 and *E. coli* S15 at the translation level, thereby highlighting the similarity between mechanisms for S15-mediated autogenous control in *E. coli* and mycobacteria. According to the mutagenic analysis, a pseudoknot in the 5′UTR of the *Msm* mRNA is strictly required for regulation.

## 2. Results and Discussion

### 2.1. A Strategy for Using Escherichia coli as a Host for Studying the Autogenous Regulation of Mycobacterial r-Proteins

The post-transcriptional control of gene expression in Actinobacteria, including protein- or sRNA-mediated riboregulation, has been poorly investigated. Our main goal was to study the autogenous control of r-protein synthesis in mycobacteria. We started with the *rpsO* gene, which was shown to be negatively regulated by its product, r-protein S15, in a range of different bacterial species (see Introduction). To study the in vivo regulation of *rpsO* from *M. smegmatis* (*Msm*), a fast-growing nonpathogenic model of *M. tuberculosis* (*Mtb*), we first applied an approach based on chromosomally integrated *rpsO’*-‘*lacZ* reporters by using *E. coli* as a host. This methodology included the creation of the *rpsO’-*‘*lacZ* reporter under the control of the *Msm rpsO* regulatory region on the plasmid pEMBLΔ46 [23] and its subsequent transfer onto the chromosome of a specialized *E. coli* strain ENS0 [23] by homologous recombination in order to provide a stable expression from a single-copy reporter gene. The use of *E. coli* as a host has previously been exploited to study the autogenous control of several r-protein operons from the γ-proteobacteria [6,8,24,25], and of the *rpsO* gene from *B. stearothermophilus* [16], but its applicability to bacterial phyla with a high GC content has not yet been corroborated. Given that the transcription and translation machineries of *E. coli* and mycobacteria have both common and significantly divergent features [26,27,28,29,30,31,32,33], it was difficult to predict in advance whether the expression of a certain mycobacterial gene in *E. coli* would be effective, as described in [29], or not. This needed to be experimentally verified.

### 2.2. Comparison of Regulatory Regions of Mycobacterial and E. coli rpsO Genes

**Figure 1:**
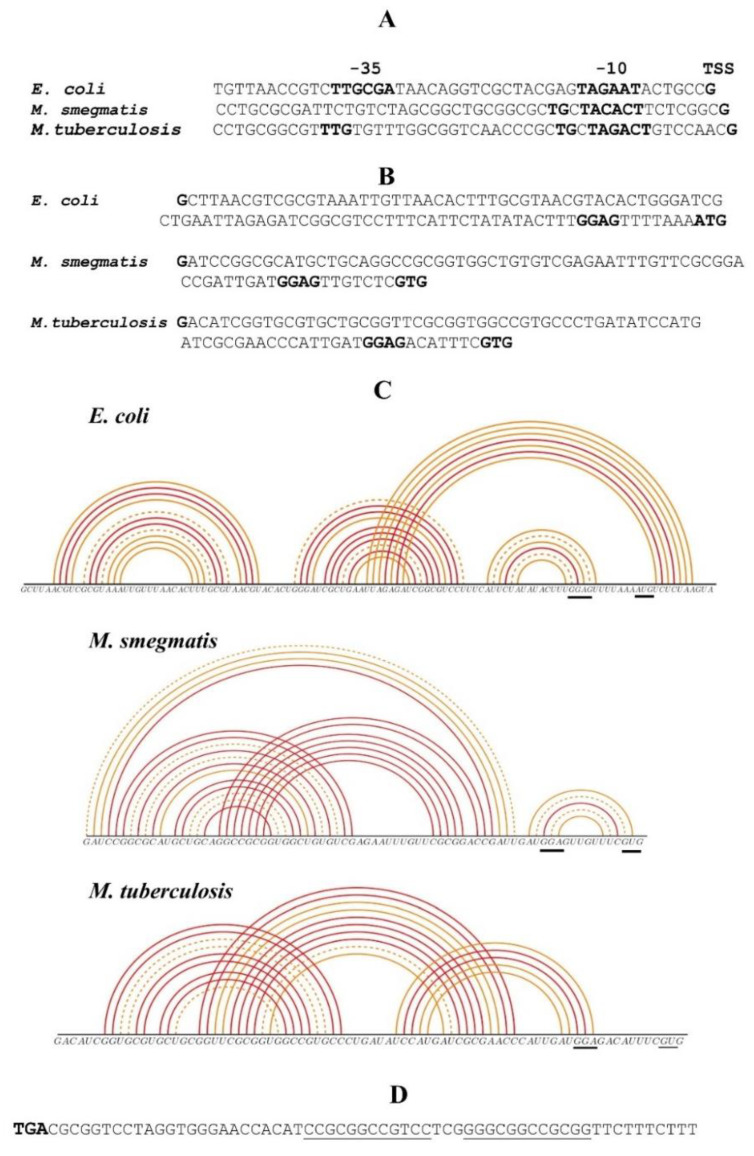
Regulatory elements in front of the *rpsO* coding region in *E. coli*, *M. smegmatis,* and *M. tuberculosis.* (**A**,**B**) Comparison of the core *rpsO* promoters (**A**) and 5′UTRs (**B**). The TSS (transcription start site), −10, −35 promoter regions (**A**) as well as the initiator codon and the Shine–Dalgarno element (**B**) are in bold. (**C**) 5′UTRs of *E. coli*, *Msm,* and *Mtb rpsO* mRNAs are able to form pseudoknots (according to [37]); the most stable structures are shown as pairing probability arcs. (**D**) Presumable intrinsic transcription terminator of the *Msm rpsO* gene; complementary sequences forming a hairpin structure are underlined.

The promoter and translation initiation regions (TIRs) of both *M. smegmatis* (*Msm*) and *M. tuberculosis (Mtb) rpsO* genes resemble those of *E. coli*, though some details appear to be quite different (Figure 1A,B). While the promoter element −10 (consensus TANNNT) is present in mycobacterial *rpsO*, the consensus region −35 is not readily defined, which is typical of mycobacterial promoters [34,35]. At the same time, both *Msm* and *Mtb rpsO* promoters belong to the class of the extended −10 promoters (TGnTANNNT), which are recognized by *E. coli* RNA polymerase [26,36], albeit the absence of the conserved TTG in the region −35 (*Msm*) may have a negative impact on the promoter activity [36]. The initiator codon is a GUG in both the *Msm* and *Mtb rpsO*, while the *rpsO* coding region starts with an AUG in *E. coli*. It is known that a GUG is used more often in mycobacteria than in *E. coli* [34], but given that several *E. coli* genes (e.g., *rpsM* encoding the r-protein uS13) show a high expression level with a GUG start codon, a combination of the *rpsO* GUG with a canonic Shine–Dalgarno (SD) element (GGAG in *Msm*, *Mtb,* and *E. coli*) may be estimated as recognizable by the *E. coli* ribosome during translation initiation.

It should be emphasized that the 5′ UTRs of *E. coli rpsO* as well as both mycobacterial *rpsO* 5′UTRs are able to fold into pseudoknots (Figure 1C). The main difference is that in *E. coli*, the pseudoknot includes the beginning of the coding sequence, while in *Msm* and *Mtb,* it is likely formed upstream of the start codon, according to the McGenus algorithm [37]. In *E. coli*, the pseudoknot in the *rpsO* TIR is recognized by uS15 and serves as an autogenous operator, providing the S15-mediated ribosome entrapment in a non-productive initiation complex [9,10,11,14]. It would be interesting to ascertain whether the mycobacterial *rpsO* pseudoknots could act in a similar way. Finally, the *Msm rpsO* operon bears an *E. coli*-like intrinsic transcriptional terminator, represented by a strong hairpin-loop structure followed by a U-rich stretch (Figure 1D), which is very convenient for constructing the plasmid for the ectopic expression of the *Msm rpsO* in *E. coli*. Thus, at first sight, one can expect that the regulatory *rpsO* regions of *Msm* or *Mtb*, including the promoter and TIR, can be recognized by the transcription/translation machineries of *E. coli*. However, the actual situation appeared to be different.

### 2.3. The rpsO Promoter from M. smegmatis Is Inoperative in E. coli

**Figure 2:**
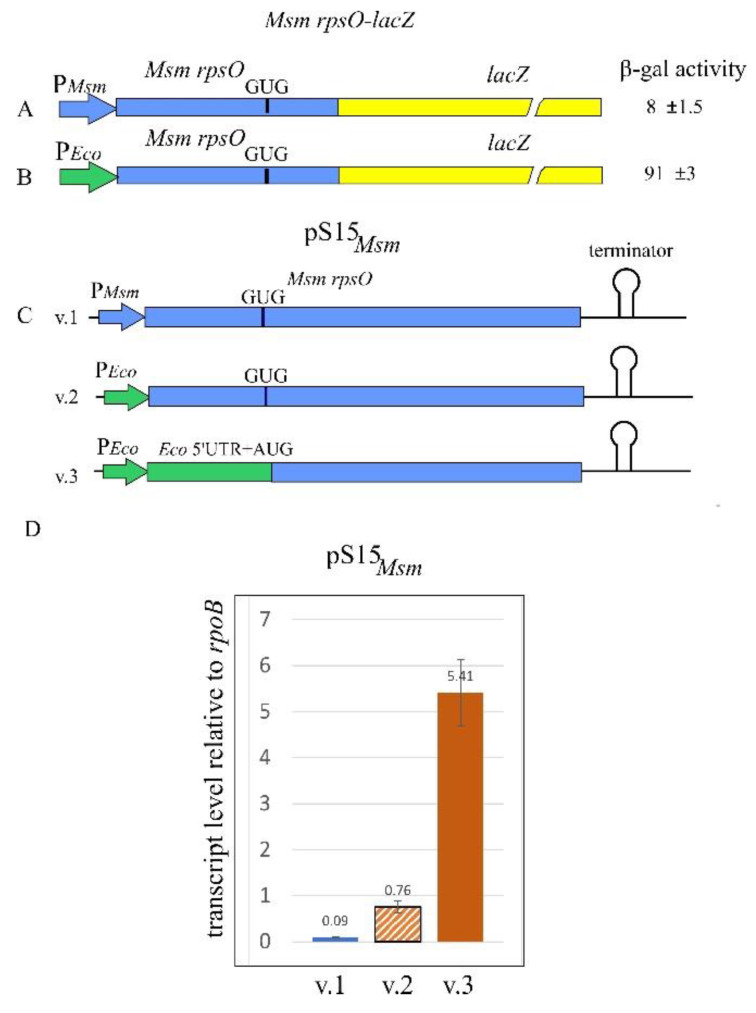
Constructs for studies of the *Msm rpsO* autoregulation by using *E. coli* as a host. (**A**) *Msm rpsO’-‘lacZ* fusion under the *Msm rpsO* promoter. (**B**) *Msm rpsO’-‘lacZ* fusion under the *E. coli rpsO* promoter. Corresponding β-galactosidase activities are indicated. (**C**) Three versions of pS15*_Msm_* plasmid (v.1, v.2, v.3) for the expression of *Msm* S15 *in trans*. (**D**) *Msm rpsO* transcript levels in *E. coli* cells bearing three versions of pS15*_Msm_*. The results of RT-qPCR analysis, with *rpoB* as an internal control. Transcript amounts relative to *rpoB* are indicated above the bars.

Following the strategy described above, we constructed the chromosomally integrated reporter *Msm rpsO’-‘lacZ* under the control of the *Msm rpsO* promoter and the TIR (Figure 2A). Although we managed to obtain the Lac^+^ phenotype resulting from homologous recombination, the β-galactosidase assay showed a very low expression output that was insufficient for statistically reliable measurements. To increase the expression level, we exchanged the *Msm rpsO* promoter for the promoter of *E. coli rpsO* while preserving the *Msm rpsO* 5′UTR intactness, which is indispensable for studying *Msm rpsO* autogenous regulation. The resulting construct showed a ca. 10-fold higher expression level (Figure 2B), thus allowing us to evaluate the impact of *Msm* S15 *in trans* on the *Msm rpsO*-*lacZ* expression.

To find the optimal construct for the *Msm rpsO* ectopic expression, we generated three versions of the plasmid pS15*_Msm_* (Figure 2C). First, using pACYC184, we cloned the whole gene *Msm rpsO* with its native flanks, including the promoter, 5′UTR, and the transcription terminator (version 1); in version 2, the *Msm rpsO* promoter was exchanged for the *E. coli* counterpart, with the *Msm rpsO* 5′UTR remaining intact; finally, in version 3, we replaced not only the *Msm rpsO* promoter, but also the 5′UTR and the initiator GUG with the *E. coli rpsO* promoter, 5′UTR, and the initiator AUG (Figure 2C).

The efficiency of the *Msm rpsO* gene expression from the constructed plasmids (pS15*_Msm_* versions 1, 2, and 3) was evaluated by measuring the *Msm rpsO* transcript level in *E. coli* cells by using RT-qPCR, with the *rpoB* transcript serving as an internal standard (Figure 2D). The highest level of the *Msm rpsO* transcript was found in cells bearing pS15*_Msm_* v.3, where the synthesis of *Msm* S15 was driven by the regulatory regions of the *E. coli rpsO*. The plasmid pS15*_Msm_* v.1 (*Msm rpsO* promoter, *Msm rpsO* 5′UTR) showed the lowest transcript yield (Figure 2D), in line with the low expression output of the *Msm rpsO’-‘lacZ* reporter. More importantly, the change of only the *Msm rpsO* promoter for the *E. coli* counterpart (pS15*_Msm_* v.2) significantly increased the transcript level, suggesting that the mycobacterial *rpsO* promoter is inoperative in *E. coli*.

Interestingly, not only the promoter region but also the 5′UTR structure had a significant impact on the transcription output. A comparison of the *Msm rpsO* transcript levels for cells bearing pS15*_Msm_*v.2 and pS15*_Msm_*v.3 revealed a seven-fold increase, indicating that the transcription and hence overall expression of the GC-rich *Msm rpsO* coding sequence become more efficient with the *E. coli* 5′UTR, despite the presence of the same *E. coli rpsO* promoter (Figure 2D). We suppose that the cognate *E. coli rpsO* TIR provides much more effective ribosome loading during translation initiation, thereby ensuring the efficient transcription–translation coupling necessary for the synthesis of a stable transcript ([38,39] and references therein). Furthermore, it has been shown that the r-protein bS1 plays a key role in the recognition and binding of mRNA 5′-UTRs by the *E. coli* 30S ribosomal subunit during initiation complex formation [40,41], including the structured *rpsO* mRNA, forming a pseudoknot that should be melted by bS1 in order to accommodate the *rpsO* TIR on the 30S ribosome [42]. As shown recently, the ability of bS1 to unfold pseudoknots inversely correlates with their structural stability [43]. This may suggest that the *Msm rpsO* 5′UTR able to form a stable pseudoknot (Figure 1C) represents an arduous target for *E. coli* S1. In addition, it has been demonstrated that the *E. coli* S1 capacity to recognize 5′UTRs of high GC-mRNAs is limited, so that the high GC content of heterologous mRNAs presents a significant challenge to *E. coli* ribosomes when initiating translation [44]. This was supported by the directed evolution of *E. coli* S1, resulting in the selection of S1 mutants capable of enhancing the translation of GC-rich mRNAs by *E*. *coli* ribosomes [44]. We suppose that the limited ability of *E. coli* S1 to recognize and unfold structured GC-rich sequences within 5′UTRs is most likely one of the main reasons behind the low expression level of the *Msm rpsO* mRNA in *E. coli*.

### 2.4. The Msm rpsO’-‘lacZ Reporter Is Regulated by Both Msm and E. coli S15

**Figure 3:**
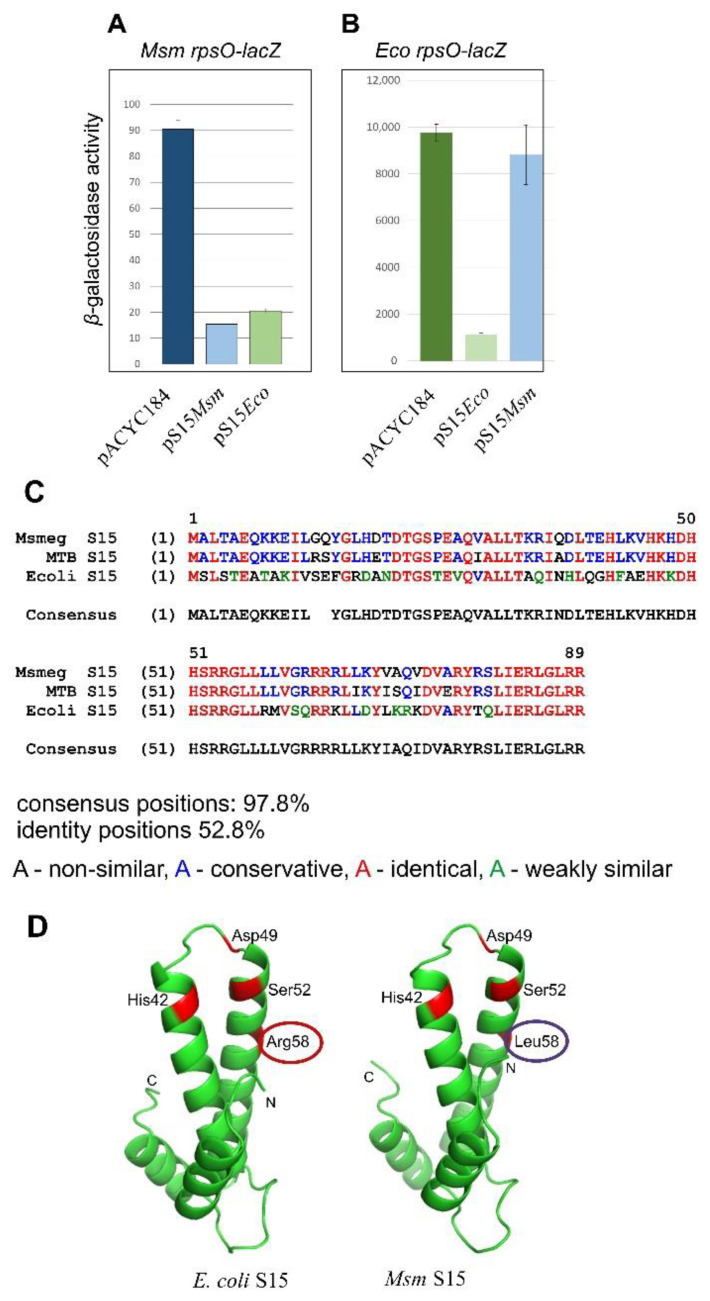
The *Msm rpsO* gene is feedback-regulated at the translation level, similar to *E. coli rpsO*. (**A**) Inhibition of the *Msm rpsO-lacZ* reporter expression in the presence of *Msm* S15 and *E. coli* S15 *in trans*. (**B**) Autogenous regulation of the *E. coli rpsO-lacZ* reporter: *E. coli* S15 *in trans* inhibits expression, while *Msm* S15 only has a marginal effect. (**C**) Alignments of S15 sequences from *E. coli*, *Msm,* and *Mtb*. (**D**) Three-dimensional structures of free S15 from *E. coli* and *Msm* as predicted by IntFOLD [45]; amino acid residues reported to be involved in mRNA binding by *E. coli* S15 [13] are shown.

Based on the above observations (Figure 2D), the plasmid pS15*_Msm_*v.3 was chosen for subsequent studies of the *Msm rpsO* autogenous control. The *E. coli* cells bearing the *Msm rpsO’-‘lacZ* reporter under the control of the *E. coli rpsO* promoter (Figure 2B) were transformed with the pS15*_Msm_*v.3 or with an empty vector, and the β-galactosidase levels were measured in transformants. Although the expression of the reporter was not high, the use of five or more biological replicates allowed us to obtain statistically reliable results which revealed ca. six-fold repression in the presence of pS15*_Msm_*v.3, thus clearly indicating the feedback regulation of the *Msm rpsO* mRNA (Figure 3A). The repression level was about the same as that for *E. coli rpsO* in the presence of pS15*_Eco_*, even though the expression of the *Eco rpsO-lacZ* reporter was incomparably higher (Figure 3B).

**Figure 4:**
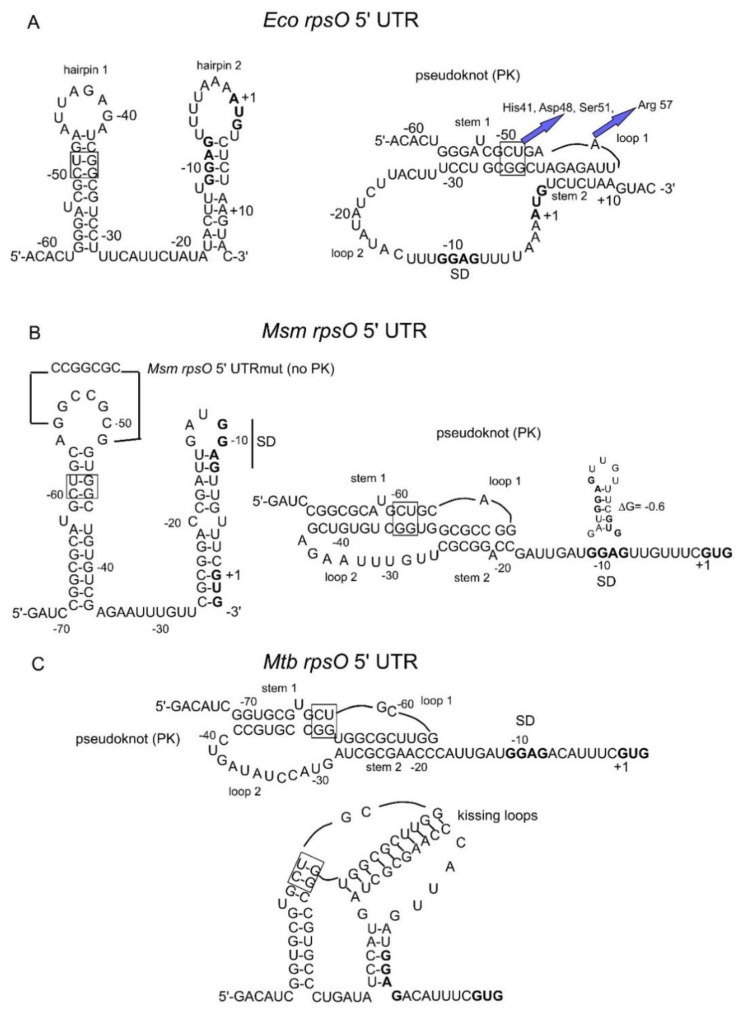
Comparison of the well-studied operator structure for *E. coli rpsO* (**A**), with predicted *rpsO* 5′UTR structures for *Msm* (**B**) and *Mtb* (**C**). The style of presentation is adopted from [46] as it allows for the revelation of more obvious similarities and differences in the RNA folds. Initiator codons and SD sequences are in bold, and a conserved U-G/C-G motif is framed. (**A**) Two topologically distinct conformations of the *E. coli rpsO* regulatory site—two stem-loops (hairpins 1 and 2) and a pseudoknot. The uS15 recognizes two sites in the pseudoknot (shown by blue arrows); respective amino acid residues involved in recognition according to [13] are indicated. (**B**) Two predicted conformations of the *Msm rpsO* 5′UTR (two hairpins and a pseudoknot) have a strong resemblance to the *E. coli rpsO* 5′UTR structures. Changes able to prevent the pseudoknot formation are shown above hairpin 1. A small hairpin shown above the ribosome binding site was predicted to exist in the most stable variant of the *Msm rpsO* pseudoknot structure (Figure 1C). (**C**) Predicted secondary structures for the *Mtb rpsO* 5′UTR (see Figure 1C): a pseudoknot bears resemblance to the *Msm rpsO* pseudoknot but may become more compacted by forming a structure of “kissing loops”.

Intriguingly, pS15*_Eco_* was also able to inhibit the expression of the *Msm rpsO’-‘lacZ* reporter, with the repression level being a little lower (Figure 3A). At the same time, pS15*_Msm_*v.3 only had a marginal impact on the expression of *Eco rpsO’-‘lacZ* (Figure 3B), indicating that despite a high homology level (Figure 3C), *Msm* S15 is not capable of recognizing the *E. coli rpsO* operator, whereas *E. coli* S15 has the ability to bind the heterological *rpsO* 5′UTR and to inhibit translation. To find out the underlying cause for this effect, we compared a set of amino acid residues for *E. coli* S15 reportedly involved in the recognition of the operator site (a pseudoknot) with residues of *Msm* S15 in the same positions (Figure 3D). It is well-established that *E. coli* S15 recognizes two sites on the pseudoknot, a U-G/C-G motif in stem 1 and the A-46 in loop 1 (Figure 4A), which are equally essential for the feedback regulation [11,12,13]. The U-G/C-G motif is recognized by the His42, Asp 49, and Ser52 of *E*. *coli* S15, while Arg58 is strictly required for the A-46 recognition, so that its exchanges for other amino acid residues impair autogenous regulation [13].

It should be mentioned that in a paper by Mathy et al. [13], the numbering of amino acid residues is different (His41, Asp48, Ser51, Arg57), as Met1 is not counted because it splits off after protein synthesis. Since we have no information about the fate of the first methionine in mycobacterial proteins, we counted all residues, including Met1 on Figure 3D. As one can see, *Msm* S15 has the same set of amino acids involved in the binding of the U-G/C-G motif, but it possesses Leu58 instead of Arg58. This difference might explain the absence of the inhibitory effect of *Msm* S15 on the *E. coli rpsO-lacZ* expression (Figure 3B).

More importantly, just as the *E. coli* regulatory region, the *Msm rpsO* 5′ UTR can be folded in two topologically distinct conformations—two stem-loops and a pseudoknot (Figure 4A,B). In *E. coli*, uS15, acting as a repressor, recognizes only a pseudoknot. By comparing the structure predicted for the *Msm rpsO* 5′UTR with the well-established *E. coli* pseudoknot, a striking resemblance is clearly visible (Figure 4A,B), including the A in loop 1. Although the *Msm* loop 2 that bridges the two stems is shorter than the *E. coli* loop 2, it is long enough (10 nts) to be recognized by *E. coli* S15, as it was shown that loop 2 could be reduced without a loss of the regulation, but not below nine nucleotides [12]. Given the visible analogy to the well-studied S15-mediated translational regulation in *E. coli*, we assume that *Msm* S15 has an ability to inhibit its own translation through a feedback regulatory mechanism at the translation level, such as that in *E. coli*.

### 2.5. Creation of the Cognate System for Studying the Autogenous Control of r-Protein Synthesis in Mycobacteria

The low expression output of the *Msm rpsO’-‘lacZ* reporter revealed obvious limitations of using *E. coli* as a host to study the autogenous control of mycobacterial r-proteins. Indeed, to enable the constructs to provide a measurable efficiency of the reporter integrated in the chromosome, we had to exchange the mycobacterial *rpsO* promoter for the *E. coli* counterpart. Furthermore, to provide the efficient expression of *Msm* S15 *in trans*, we had to substitute the regulatory region, including the promoter 5′UTR and the start codon, for respective *E. coli* determinants. In addition, as uS15 is a small protein (89 amino acid residues), the expression of the *Msm rpsO* short coding region in *E. coli* did not appear to be a very difficult task for the transcription/translation machineries of *E. coli* even though they had been adapted to a lower GC content. It is reasonable to suspect that the mycobacterial mRNAs encoding longer r-proteins (such as RpsA, RpsB) will bring far more problems, making the use of *E. coli* as a host unpromising for future studies. Thus, it is vital to develop the authentic system for studies of the r-protein-mediated control in mycobacteria, and *M. smegmatis* represents the best proxy for such experiments [21].

**Figure 5:**
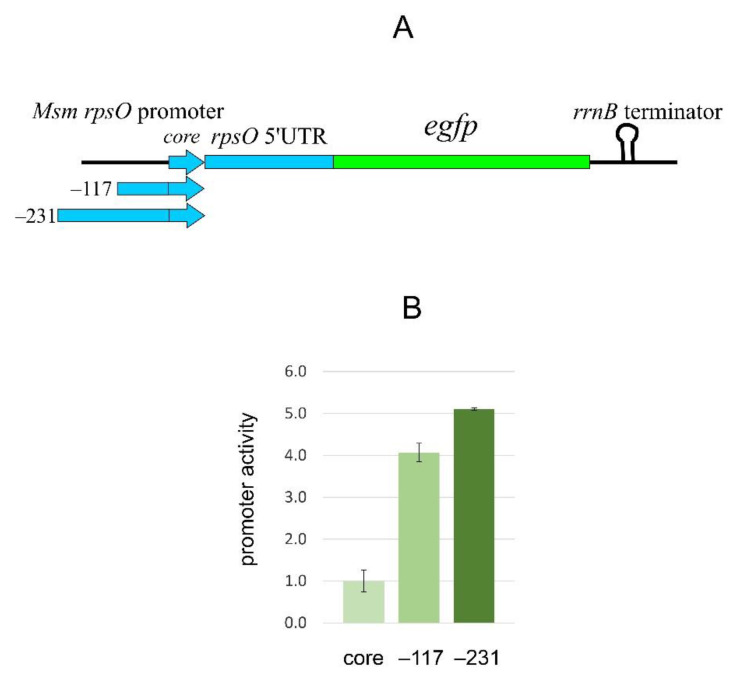
The expression level of the chromosomally integrated *Msm rpsO-egfp* reporter depends on the 5′-extension of the *Msm rpsO* promoter. (**A**) *Msm rpsO-egfp* reporters with different 5′ extensions within the *Msm* chromosome. (**B**) Results of fluorescent measurements of protein lysates obtained from exponential *Msm* cells bearing *rpsO-egfp* reporters governed by the *rpsO* promoters with different 5′ extensions; positions of relative TSS are indicated, with “core” corresponding to the 5′ edge position −47. Fluorescence (average of at least three biological replicates) of the protein samples corresponding to the core promoter construct is taken as a unit.

Both integrative (to be inserted into mycobacterial chromosome) and replicative (for ectopic expression of genes under study) plasmids for creating the *Msm*-based reporter system were reported [22] and widely used. The integrative plasmid pMV306hsp was initially derived from a replicative vector pMV261 by replacing the mycobacterial replication origin (*oriM*) with a DNA fragment comprising the attachment site *attP* and the integrase gene *int* from the mycobacteriophage L5, which provided a site-specific integration into the chromosomal *attB* site [22]. In addition, this vector carries the *hsp*60 promoter and the *rrnB* terminator to facilitate the cloning and expression of different genes as a single copy integrated into the chromosome. We modified pMV306hsp by replacing the region comprising the *hsp* promoter with the *Msm rpsO-egfp* reporter bearing the *Msm rpsO* core promoter and 5′UTR in front of the eGFP coding sequence, so that the transcription of the reporter gene would be governed by the *rpsO* core promoter and terminate at the *rrnB* terminator (Figure 5A).

### 2.6. The Msm rpsO Core Promoter Requires an Upstream Region to Enhance the Transcription Yield

To evaluate the efficiency of the chromosomally integrated fluorescent reporter, the *Msm* cells (kan^r^) were harvested at the exponential phase (OD_600_~0.7–0.8) and then disintegrated for the preparation of protein lysates to be used for measuring the fluorescence of the reporter. The fluorescence appeared unexpectedly low despite the fact that ribosomal core promoters, at least in *E. coli*, are generally effective (e.g., the *rpsO* promoter in a fusion *Eco*_*rpsO-lacZ*, see Figure 3B). An analysis of the published data revealed that, in contrast to *E. coli,* mycobacterial core promoters (including only −10 and −35 promoter regions) may be inefficient, requiring 5′extensons to augment their strength [27,47,48]. In particular, the core *Msm rrnB* promoter (which a priori should be one of the strongest in bacterial cells) remained relatively weak unless and until the upstream region was significantly extended [27]. To test whether it is also the case with the *rpsO* gene, we extended the *rpsO* promoter sequence (the initial 5′ edge was at position −47 from TSS) to obtain the 5′ extended variants: version 2 (−117) and version 3 (−231), and then created the *Msm* cells bearing the corresponding *rpsO-egfp* reporters in the chromosome (Figure 5A). The fluorescence measurements revealed the increased yield of eGFP in the extended promoter variants (Figure 5B). The same was previously shown for the *Msm rrnB* and Ms1 promoters [27,47]. The exact mechanism for the enhancement of transcription efficiency upon promoter extension has not yet been clarified. The *Msm rpsO* gene is transcribed from a single promoter [49,50]; hence, the impact of additional upstream promoters on transcription yields is unlikely. One of the reasonable explanations is the existence of upstream binding sites for yet unknown transcription factors acting as activators [27,47]. Based on the experimental observations, we used the *Msm rpsO*-*egfp* fusion bearing the −231 extension for further experiments.

### 2.7. Generation of the Novel Replicative Shuttle Vector, pAMYC

**Figure 6:**
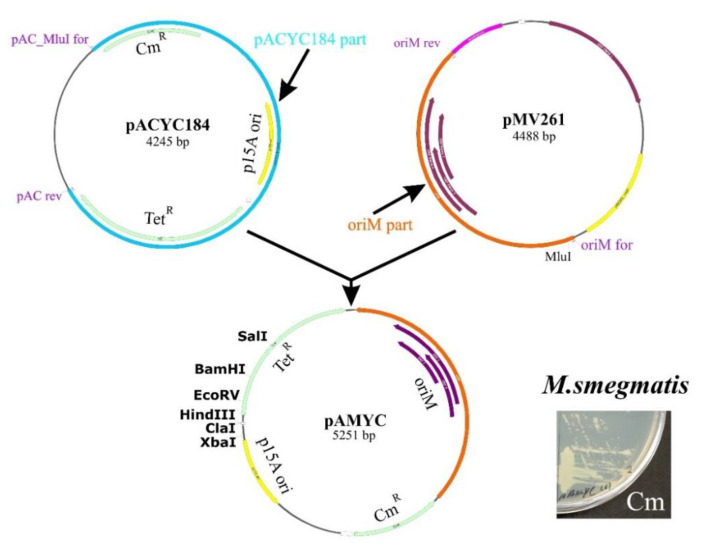
The scheme for creating the novel replicative shuttle vector, pAMYC, a derivative of pACYC184. Transformation of *M. smegmatis* by pAMYC results in the appearance of chloramphenicol-resistant colonies on LB-Cm plates.

To provide the ectopic expression of the *Msm rpsO* gene necessary to study the S15-mediated effect on the efficiency of the *rpsO-egfp* reporter, we created a novel shuttle replicative vector, pAMYC, by transferring the region comprising the mycobacterial replication origin from pMV261 to pACYC184 (Figure 6). The vector pMV261 itself is not applicable because it bears the same kanamycin-resistant marker as an integrative pMV306 used for the incorporation of the *rpsO-egfp* fusion into the *Msm* chromosome. The electroporation of the novel shuttle plasmid pAMYC into *Msm* cells yielded chloramphenicol-resistant transformants, indicating its suitability for the ectopic expression of different mycobacterial genes (Figure 6).

### 2.8. Mycobacterial rpsO Expression Is Feedback-Regulated at the Translation Level

**Figure 7:**
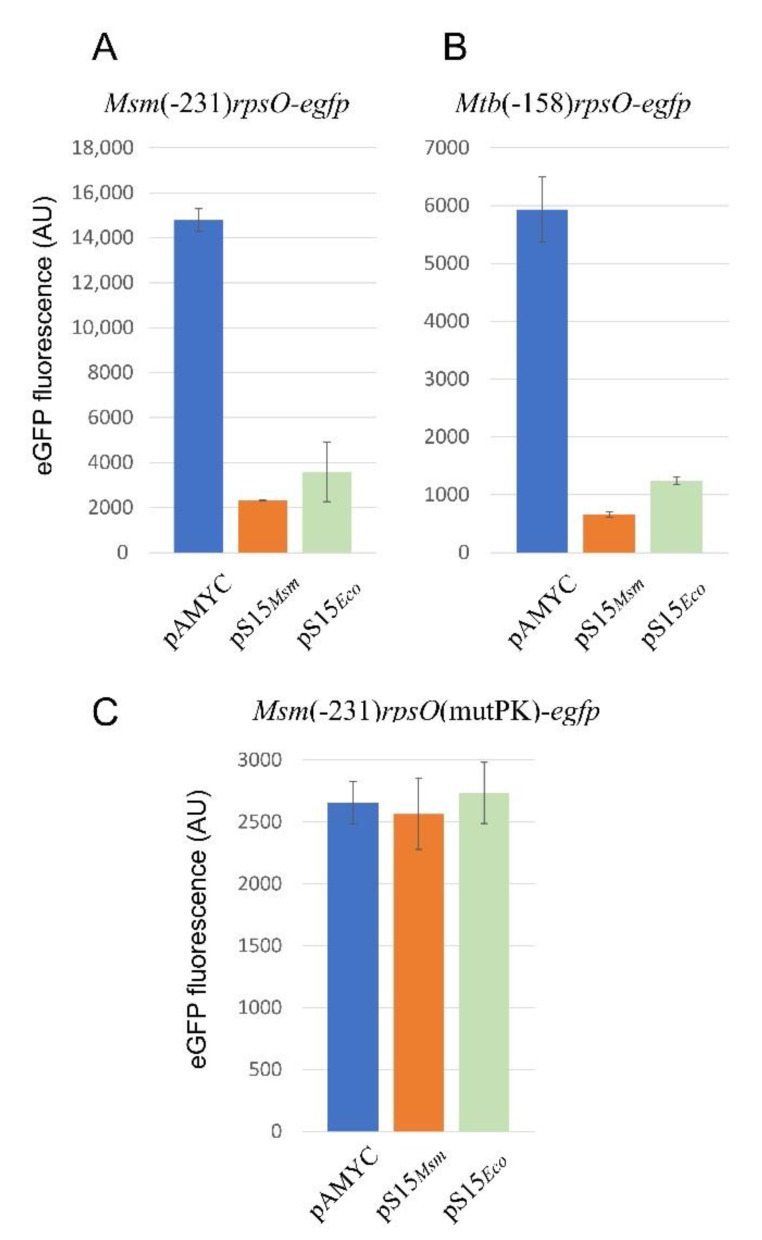
The *rpsO* genes of *M. smegmatis* and *M. tuberculosis* are feedback-regulated in vivo at the translation level. Repression of the *Msm rpsO-egfp* (**A**) and *Mtb rpsO-egfp* (**B**) expression in the presence of the pAMYC derivative expressing *Msm* S15 and *E. coli* S15. Results of fluorescent measurements of protein lysates from at least three biological replicates. (**C**) The pseudoknot is crucial for the regulation of the *Msm rpsO-egfp* reporter; changes preventing its formation (see Figure 4B) abolish the feedback regulation.

To facilitate the synthesis of *Msm* S15 in trans, we cloned the *Msm rpsO* gene bearing the 5′-extended promoter variant (−231) and its own intrinsic terminator into pAMYC. The resulting plasmid pAMS15*_Msm_* was used to transform *Msm* cells bearing the chromosomal *rpsO-egfp* fusion under the same 5′-extended (−231) *rpsO* promoter. An empty pAMYC served as a control. Exponential *Msm* cells were harvested and disintegrated to prepare protein lysates where the eGFP fluorescence was measured. The results clearly showed the reduced fluorescence in cells bearing pAMS15*_Msm_* when compared to the control cells bearing an empty pAMYC (Figure 7A), thus indicating that uS15 *in trans* down-regulates the reporter expression. This strengthens the results obtained with *E. coli* as a host. Just as in *E. coli* (Figure 3A), *Msm* S15 *in trans* also had an inhibitory effect on the *Msm rpsO-egfp* expression (Figure 7A).

Furthermore, we created an analogous, chromosomally integrated reporter where the eGFP expression was under the control of the extended (−158) promoter and the 5′UTR of the *Mtb rpsO* gene. The fluorescence was measured for the *Msm* cells bearing the pAMYC derivative expressing *Msm* S15 (which is 88.8% identical to the *Mtb* S15) or *E. coli* S15, and an empty vector as a control. The results clearly showed the repression of the *Mtb rpsO-egfp* expression by both *Msm* S15 and *E. coli* S15 in trans (Figure 7B). Taken together, the results allow us to conclude that mycobacterial *rpsO* expression is feedback-regulated at the translation level, and that the mechanism likely resembles that of *E. coli*. Most probably, acting as a repressor, uS15 binds the 5′UTRs of the *Msm* and *Mtb rpsO* mRNAs folded into pseudoknots (Figure 4B,C), thereby stabilizing its structure and impeding ribosome loading to initiate translation. For instance, the *Mtb* 5′UTR may be stabilized by S15 in a compacted “kissing loops” structure, which hides the SD sequence from ribosome binding (Figure 4C).

### 2.9. The Pseudoknot in the Msm rpsO 5′UTR Is Essential for the Autogenous Control

To obtain direct proof of the essential role of pseudoknot formation in the feedback control of the *Msm rpsO* expression, we mutagenized the sequence involved in the pseudoknot by exchanging the GGCCGCG for the CCGGCGC (Figure 4B). This should destroy the stem 2 in a pseudoknot, such that the 5′UTR could only form a double hairpin conformation. The mutated variant of the *Msm rpsO-egfp* (mutPK) reporter was incorporated into the *Msm* chromosome, and fluorescence was measured in the corresponding cells in the presence of *Msm* S15 or *E. coli* S15 *in trans* vs. an empty vector. The data obtained (Figure 7C) clearly show the total absence of the S15-mediated repression (by both *Msm* S15 and *E. coli* S15) accompanied by reduced expression efficiency (compare Figure 7A and Figure 7C). This suggests that a pseudoknot structure in the 5′UTR of the *Msm rpsO* mRNA is preferentially recognized both by S15 as an autogenous repressor and by the ribosome during translation initiation. The mutated *Msm rpsO* 5′UTR in a double harpin conformation hides the initiator GUG and partly the SD-sequence from ribosome recognition (Figure 4B), thereby reducing the efficiency of translation initiation. *E. coli* S15 acts largely in a similar way (Figure 7C), thus allowing us to conclude that the mycobacterial autogenous control of the *rpsO* expression bears close resemblance to that of *E. coli* despite the large phylogenetic distance between Gram-negative gamma-proteobacteria and high GC Gram-positive mycobacteria.

### 2.10. Concluding Remarks

In this work, we provided evidence that the autogenous control of r-protein synthesis at the translation level functions in mycobacteria. We developed a reporter system based on *M. smegmatis,* which is suitable for the study of the regulation of mycobacterial genes that encode r-proteins. The use of *E. coli* as a host for this purpose was found unpromising because of the low efficiency of the *E. coli* transcription/translation machinery in the expression of mycobacterial genes. To study the regulation of the *rpsO* gene encoding r-protein S15, we obtained the *Msm* cells bearing the reporters *Msm_rpsO-egfp* and *Mtb_rpsO-egfp* in the chromosome and measured their activity in the presence of *Msm* S15 expressed from the new replicative shuttle plasmid pAMYC vs. an empty vector. The inhibition of the reporter expression by *Msm*S15 *in trans* clearly indicated the autogenous control of the *rpsO* expression in mycobacteria.

The most important finding is that the autogenic regulation of the mycobacterial *rpsO* genes strictly required the pseudoknot conformation of the 5′UTR, so that the mutagenesis of the sequence involved in the formation of the pseudoknot completely abolished the S15-mediated repression. Moreover, *E. coli* S15 was also found to be capable of acting as a repressor of the *Msm/Mtb rpsO* expression, and this ability was lost when the pseudoknot structure within the 5′UTR was destroyed by mutations. This provides evidence that the mechanism for the S15-mediated autogenous control in mycobacteria bears close resemblance to that described for *E. coli* despite the large phylogenetic distance between these bacterial species. In other words, the involvement of the pseudoknot in the S15-mediated autogenous regulation is not only specific for *E. coli* but may have independently emerged in distant mycobacterial species. 

## 3. Materials and Methods

### 3.1. Strains and Plasmids

Strains and plasmids used in this study are listed in Table 1. *Mycobacterium smegmatis* mc^2^ 155 [51] was provided by Prof. A. S. Kaprelyants (Bach Institute of Biochemistry, Moscow, Russia). Isolation of *M. smegmatis* genomic DNAs was performed according to Belisle et al. [52]. *M. tuberculosis* genomic DNA was a kind gift from Dr. E. Salina (Bach Institute of Biochemistry, Moscow, Russia). For experiments with *E. coli* as a surrogate host, plasmids pS15*_Msm_* (versions 1, 2, 3), derivatives of the pACYC184 cloning vector, were constructed to express *in trans* the *rpsO* gene from *M. smegmatis* (*Msm*). The plasmid pEMsm_rpsO-lacZ, a derivative of pEMBLΔ46 [23] bearing the *Msm rpsO’-*‘*lacZ* fusion, was used to transfer this reporter onto the chromosome of ENS0 by homologous recombination. For the *M. smegmatis* expression system, the derivatives of the pMV306 integrative plasmid bearing the kanamycin-resistant marker [22] and the novel replicative shuttle vector pAMYC (providing chloramphenicol resistance) were created (see below).

### 3.2. Construction of Expression Plasmids for Use in E. coli as a Surrogate Host

To generate pS15*_Msm_* (v.1), the *rpsO* gene, flanked with its own promoter and terminator sequences, was amplified by PCR on *Msm* genomic DNA with the primers Msm-rpsO-for 5′-ATC***GGATCC***GCACGATCCTGC and Msm-rpsO-rev 5′-ACT***AAGCTT***GCATGTCCGCAGAC. Forward and reverse primers comprised BamHI (for) and HindIII (rev) sites (bold italicized) for subsequent cloning in pACYC184. To create pS15*_Msm_* (v.2), the *Msm rpsO* promoter was replaced with the *E. coli rpsO* promoter by using a two-step PCR technique. First, two PCR fragments were obtained; one was amplified on pS15*_Eco_* (a pACYC184 derivative bearing the *E. coli rpsO* gene flanked with its native promoter and terminator, see [53]), using the forward primer corresponding to the pACYC184 sequence, including the BamHI site (pACYC184-for 5′-CGATGCGTCCGGCGTAGA***GGATCC***) and the reverse primer (PrpsOmix-rev) comprising a sequence complementary both to the *E. coli rpsO* promoter/discriminator region and the beginning of the *Msm rpsO* transcript (5′-CATGCGCCGGATCGGCAGTATTCTACTC, with the *Msm* sequence underlined). Another PCR fragment was amplified on pS15*_Msm_* (v.1) with the primers PrpsOmix-for, a complement of the PrpsOmix-rev (5′-GAG**TAGAAT**ACTGCCGATCCGGCGCATG where the *Msm* sequence is underlined, and the *Eco_rpsO* −10 promoter is in bold) and Msm-rpsO-rev, described above. Second, the two PCR fragments were mixed and amplified with the external primers pACYC184-for and Msm-rpsO-rev; the resulting product was treated with BamHI and HindIII and cloned in pACYC184/BamHI, HindIII.

Lastly, to create pS15*_Msm_* (v.3), not only the *Msm rpsO* promoter but also 5′-UTR and the start codon GUG were substituted for the corresponding *E. coli* sequences. As the first step, two PCR fragments were obtained: one amplified on pS15*_Eco_* with the primers pACYC184-for (see above) and Eco-rpsOTIR-rev (5′-CGGCGGTAAGCGCCATTTTAAAACTCCAAAG, where the *Msm* sequence is underlined), and another one amplified on pS15*_Msm_* (v.1) with the primers Eco-rpsOTIR-for (5′-CTTT**GGAG**TTTTAAA**ATG**GCGCTTACCGCCG, where *E. coli* SD-sequence and AUG start codon are in bold, and the *Msm rpsO* sequence is underlined) and Msm-rpsO-rev. As the second step, the two PCR fragments were mixed and amplified in the presence of pACYC184-for and Msm-rpsO-rev; the resulting product was cloned in pACYC184, as described above. All three versions of pS15*_Msm_* were sequenced and used for further experiments.

### 3.3. Quantification of the In Vivo Transcripts by RT-qPCR with an Internal Standard

The efficiency of the *Msm rpsO* gene expression in *E. coli* was evaluated by measuring the *Msm rpsO* transcript levels in cells bearing plasmids pS15*_Msm_* (versions 1, 2, 3) or the parental empty vector pACYC184 as a control. Total RNA was isolated by using the RNeasy mini kit (Qiagen, Hilden, Germany) according to recommendations of the manufacturer. Strains (including the control bearing an empty vector) were grown in LB medium at 37 °C, with vigorous shaking. At an optical density of 600 nm (OD_600_) of ~0.4, 2 mL aliquots of cell cultures were withdrawn and mixed with a 4 mL RNAprotect bacterial reagent. Next, total RNA was extracted; during extraction, RNase-free DNase was added to the columns for 15 min to eliminate DNA contaminations in RNA samples; after elution, RNA concentrations were estimated by measuring the OD_260_. Reverse transcription (RT) was performed with AMV reverse transcriptase (Promega Corporation, Madison, USA) in the final volume of 20 μL for 1 h at 42 °C on 1 μg of the total RNA in the presence of two reverse primers (1 μL of 5 μM solution each) corresponding to the coding part of *Msm rpsO* (Msm-rpsOcod-rev 5′-CGAGTGGTGATCGTGCTTGTGC) and to the reference gene *rpoB* (rpoB-rev: 5′-CGGATTTGACATTCCTGGACGTC). Real-time PCR (qPCR) was run with the use of LightCycler 96 (Roche, Basel, Switzerland); each 25 μL reaction contained 2 μL of the RT mix, 5μL 5x qPCRmix HS SYBR (Evrogen, Moscow, Russia), forward primers corresponding to the beginning of transcripts (Msm-rpsOtr-for 5′-GTGGCTGTGTCGAGAATTTGTTCG for pS15*_Msm_* v.1 and 2, Eco-rpsOtr-for 5′-GTAACGTACACTGGGATCGCTG for pS15*_Msm_* v.3, rpoB-for 5′-ACGTCCACAAGTTCTGGATGTACC) and reverse primers used for RT (1 μL of a 5 µM solution each). Two independently isolated preparations of total RNA for each of the 4 strains were used for RT, and three technical replicates for each qPCR reaction were run simultaneously. Control qPCR reactions without RT were performed to exclude DNA contaminations in RNA preparations. LinRegPCR software was used to quantify transcript amounts relative to the reference transcript *rpoB*.

### 3.4. Construction of the Msm_rpsO’-‘lacZ Fusions Integrated into the E. coli Chromosome

The *Msm_rpsO’-‘lacZ* chromosomal fusions were generated, as previously described for different fusions related to the r-protein operons from γ-proteobacteria (see, e.g., [24,25]). For the fusion under the control of the *Msm rpsO*-promoter, a DNA fragment was amplified on pS15_Msm_v1 with the primers Msm-rpsO-for (see above) and Msm_TIR_rpsOrev 5′-GG*AAGCTT*TGGCCCAGGATCTC. The forward primer comprised the BamHI site, the reverse primer—HindIII (italicized in the sequence). The resulting fragment was cloned in pEMBL Δ46/BamHI, HindIII in frame with the *lacZ* open reading frame, then the correct construct was transferred onto the chromosome of ENS0 (Table) by homologous recombination, followed by the selection of the recombinant Lac^+^ strains on McConkey agar. To substitute the *Msm rpsO* promoter with the *E. coli* counterpart, the corresponding DNA fragment was amplified on pS15_Msm_v2 with the primers pACYC184-for and Msm_TIR_rpsOrev (see above), cloned in pEMBLΔ46/BamHI, HindIII, and then transferred onto the chromosome of ENS0, as described above.

### 3.5. Cell Growth and β-Galactosidase Assay

*E. coli* cells bearing the *Msm rpsO’-‘lacZ* reporters and the plasmid expressing *Msm* S15 or the empty vector were grown at 37 °C in Luria-Bertani (LB) medium supplemented with chloramphenicol (34 μg/mL), harvested in exponential phase at OD_600_ ~ 0.4–0.5, and used for the preparation of clarified cell lysates, essentially as described in [24]. The protein concentration in each fraction of the soluble proteins was determined by Bradford assay (Bio-Rad, Hercules, California, USA). Specific ß-galactosidase activities in the same fractions were measured according to Miller [55] and expressed in nmol ONPG (*ο*-nitrophenyl-β-D-galactopyranoside), hydrolyzed per minute per milligram of total soluble cell proteins.

### 3.6. Creation of the Novel Escherichia coli-Mycobacteria Shuttle Vector pAMYC, a Derivative of pACYC184

A 3328 bp- fragment of pACYC184 comprising genes for chloramphenicol (Cm) and tetracycline (Tet) resistance as well as a replication origin p15A (*oriE*) was PCR-amplified by using Q5 High-Fidelity DNA Polymerase (New England Biolabs, Hitchin, Hertfordshire, UK) with the primers pACshtl- for (5′-TTC*ACGCGT*AGCACCAGGCG, MluI restriction site italicized) and pACshtl-rev (5′-CTCCGCAAGAATTGATTGGCTCC). Mycobacterial origin of the replication (*oriM*) was amplified from pMV261 [24] by using Q5 DNA Polymerase and the primers oriM-for (5′-GCCTTTGAGTGAGCTGATACCG) and oriM-rev (5′-GATTTAAAGATCTGGTACCGCGGC), resulting in a 1976-bp PCR fragment. The PCR fragments (3328 and 1976 bp in length) were gel-purified, treated with MluI (MluI site in the *oriM*-fragment is located near the annealing site for oriM-for), phosphorylated at blunt ends by treatment with T4-PNK (Thermo Scientific, Dreieich, Germany), and then ligated by T4-DNA ligase (Thermo Scientific, Dreieich, Germany) at room temperature. Ligation mix was used to transform DH−5α cells; plasmids were isolated from Cm^r^- transformants and used for electroporation of *M. smegmatis* cells [56]. Cm-resistant colonies appeared on LB-Cm agar plates after 3 days of incubation at 37 °C, indicating that a newly created plasmid (named pAMYC) indeed works as a mycobacteria-*E. coli* shuttle vector and may thus be used for cloning and expression *in trans* of mycobacterial proteins (or sRNAs, depending on the task) to study their effect on the expression of mycobacterial mRNA targets.

### 3.7. Modification of the Integrative Plasmid pMV306hsp to Provide Insertion of the rpsO-egfp Reporter Construct into the Chromosome of M. smegmatis

The integrative shuttle vector pMV306hsp [22] carries the *hsp*60 promoter and the *rrnB* terminator to facilitate the cloning and expression of different genes as a single copy integrated into the chromosome. This plasmid was modified by deleting the region comprising the *hsp60* promoter and inserting the *rpsO_Msm_*-e*gfp* reporter in front of the *rrnB* terminator. To this end, pMV306hsp was treated with endonucleases MluI (upstream of the *hsp* promoter) and HindIII (in front of the *rrnB* terminator), and then dephosphorylated with TSAP (Thermosensitive Alkaline Phosphatase, Promega, Madison, WI, USA).

To generate inserts comprising the *egfp* reporter under the control of the *Msm rpsO* regulatory regions (including the promoter and 5′-UTR), the *rpsO-egfp* fusions were generated by the two-step PCR technique with overlapping primers. For the first fusion, the *Msm rpsO* core promoter was used, 5′-end of which corresponded to the −47 position from the transcription start site (TSS). The *rpsO* part was amplified from the *M. smegmatis* genomic DNA by using Tersus Plus PCR kit (Evrogen, Moscow, Russia) with primers (−47) rpsO-for (5′-CTA*ACGCGT*TCCTGCGCGATTCTG, MluI site italicized) and rpsO-egfp--rev (5′-CGCCCTTGCTCACCACGAAACAACTCCA). The *egfp* part was amplified from pQE30-egfp (Table), with primers rpsO-egfp-for (complementary to the rpsO-egfp-rev) and pQEegfp-rev (5′-GGAGTCCAAGCTCAGCTAATTAAGC, located downstream from HindIII site of pQE30-egfp). At the second step, the two PCR products were mixed and amplified with the external primers (−47) rpsO-for and pQE30egfp-rev, and the resulting product was cleaned from 2% agarose gel by Cleanup Standard Kit (Evrogen, Moscow, Russia), digested by MluI and HindIII, and ligated into pMV306/MluI, HindIII. The reporter constructs bearing 5′-extended *rpsO* promoters were created in a similar way with the primers (−117) rpsO-for (5′-TCT*ACGCGT*AGGAGAAGTTCGATTC) and (−231) rpsO-for (5′-TGA*ACGCGT*AATCCGACGTTCTC), while other primers were the same as described above.

The *rpsO-egfp* reporter construct bearing the 5′UTR and the *rpsO* promoter from *M. tuberculosis* (*Mtb*) was created analogously. In this case, the promoter region was 5′-extended up to position −158 from TSS. Primers used for the two-step PCR: Mtb_rpsO-for (5′- AGA*ACGCGT*TCGAATCGGTGCG, MluI site italicized), Mtb_rpsO-egfp-rev (5′-CGCCCTTGCTCACGAAATGTCTCCATC), Mtb_rpsO-egfp-for (5′-GATGGAGACATTTC**GTG**AGCAAGGGCG, initiator GUG in bold) and pQEegfp-rev (see above). All amplification reactions were performed with the Tersus Plus PCR kit (Evrogen, Moscow, Russia).

### 3.8. Mutagenesis of the Msm rpsO 5′UTR to Prevent Pseudoknot Formation

To study the potential role of a pseudoknot within 5′UTR of the *Msm rpsO* mRNA in its expression and regulation, the sequence GGCCGCG involved in the pseudoknot formation was substituted for CCGGCGC (Figure 4B). To this end, a two-step PCR technique was used. First, two PCR products were obtained on the pMV306 derivative bearing the *Msm rpsO-egfp* fusion under the extended (−231) *rpsO* promoter with two pairs of primers: P(−231)rpsO-for_MluI 5′ TGAACGCGTAATCCGACGTTCTC (MluI site underlined) with rpsO_ mutPK- rev 5′ GCGCCGGTGCAGCATGCGCCGGATCG, and rpsO_ mutPK- for 5′ CCGGCGCG GGCTGTGTCGAGAATTTG with egfp-HindIII- rev 5′ ATTAAGCTTTCACTTGTACAGCTCGTC (HindIII site underlined). -Second, the two PCR fragments were mixed and amplified in the presence of external primers P(−231)rpsO-for and egfp-HindIII- rev. The product was digested with MluI and HindIII and cloned into pMV306/MluI, HindIII. The resulting plasmid was sequenced and used to insert the mutated *rpsO-egfp* fusion (mutPK) into the *Msm* chromosome.

### 3.9. Creating Plasmids for Ectopic Expression of the Msm (or E. coli) rpsO Gene in M. smegmatis

To create the pAMYC derivative expressing *Msm* S15, the *rpsO* gene flanked with the 5′- extended promoter and terminator regions was amplified on the *Msm* genomic DNA by using Q5 DNA polymerase and primers P(−231)rpsO-for_BamHI bearing BamHI (5′-TGA***GGATCC***AATCCGACGTTCTC, BamHI in bold and italicized) and Msm-rpsO-rev (5′-ACT***AAGCTT***GCATGTCCGCAGAC, HindIII in bold and italicized). The PCR product was treated with BamHI /HindIII and then ligated into pAMYC treated with the same endonucleases. The ligation mix was used to transform *E. coli*; next, plasmids were isolated from Cm-resistant colonies, sequenced, and further used to transform *Msm* cells bearing the reporter *Msm (or Mtb) rpsO-egfp*. To create the pAMYC derivative expressing *E. coli* S15, the BamHI-HindIII fragment from pS15*_Eco_* was cloned into pAMYC.

### 3.10. Cell Growth and eGFP Assay

Transformation-proficient *M. smegmatis* mc^2^ 155 [51] was used for electroporation with pMV306 (Kan^r^) derivatives bearing the *rpsO-egfp* reporter genes to provide their insertion into the chromosome. The Kan^r^-transformants were selected on LB-Kan agar plates, and then used for competent cell preparation and electroporation with an empty shuttle vector pAMYC, or with its derivatives carrying the *Msm* (or *E. coli*) *rpsO* gene for uS15 expression *in trans* (see above). The transformants were selected on LB-Kan-Cm agar plates, then grown at 37 °C in LB supplemented with 34 μg/mL Cm and 0.05% Tween 80 (to prevent cell clumping), and then harvested in exponential phase (OD_600_ ∼0.7–0.8). Protein extracts were prepared as described in [57], with slight modifications. The cell pellets were resuspended in PBS and broken by using Beat Beater and 0.1 mm zircon beads (BioSpec Products Inc., Bartlesville, USA) (3 times for 30 s on ice, with 1:4 vol/vol ratio of beads to cell suspension). The cell lysates were clarified by centrifugation (20 min, 12,000× *g* rpm at 4 °C), supernatants were treated with RQ-DNase (Promega, Madison, WI, USA) for 30 min on ice and used for the eGFP assay. Protein concentration in clarified lysates was determined by Bradford assay (Bio-Rad, Hercules, CA, USA). The enhanced green fluorescent protein (eGFP) has an excitation peak at 488 nm (blue light) and emits light maximally at 507 nm [58]. EGFP fluorescence in protein samples was measured in a 96-well microplate using Tecan Genios Pro fluorescence microplate reader (Tecan, Switzerland) and standard excitation–emission filters. The results were normalized to the protein concentration in samples. Each sample was obtained in at least three biological replicates. As a background control, protein lysates obtained from exponentially grown *Msm* cells were used.

## Figures and Tables

**Table 1:** Strains and plasmids used in this study.

Strain/Plasmid	Relevant Characteristics	Reference or Source
Strains		
*M. smegmatis* mc^2^155		[51]
DH5a	*E. coli* cloning host	Laboratory stock
ENS0	*E. coli* strain, *his*, formerly HfrG6D12	[23]
IBrpsO188:lacZ	ENS0 bearing *E. coli rpsO’-‘lacZ*	[53]
LAB_P_Eco_rpsO_Msm_:lacZ	ENS0 bearing *Msm rpsO’-‘lacZ* under *E. coli rpsO* promoter	This paper
Msm_PrpsO_Msm_:egfp	*M. smegmatis* bearing *Msm rpsO’-egfp*	This paper
Msm_PrpsO_Msm_:egfp	*M. smegmatis* bearing *Msm rpsO’-egfp* in the chromosome (Kan^r^) under the 5’ extended *rpsO* promoter (−231)	This paper
Msm_PrpsO_Mtb_:egfp	*M. smegmatis* bearing *Mtb rpsO’-egfp* reporter in the chromosome (Kan^r^) under the *Mtb rpsO* promoter (−158)	This paper
Plasmids		
pEMBL 46	pEMBL8^+^derivative lacking *lacZ* RBS	[23]
pES15*_Msm_*TIR(v1)	pEMBL 46 derivative bearing *rpsO_Msm_’-‘lacZ* reporter under the *Msm* core *rpsO* promoter	This paper
pES15*_Msm_*TIR(v2)	pEMBL 46 derivative bearing *rpsO_Msm_’-‘lacZ* reporter under the *E. coli rpsO* promoter	This paper
pACYC184	Tet^r^, Cm^r^, cloning vector	[54]
pQE30_egfp	derivative of pQE30 (Qiagen) expressing the e*gfp* gene	Lukyanov KA#
pS15 (pS15*_Eco_*)	pACYC184 derivative expressing *E. coli rpsO*	[52]
pS15*_Msm_*(v1)	pACYC184 derivative expressing *Msm rpsO* under *Msm rpsO* core promoter and 5’UTR	This paper
pS15*_Msm_*(v2)	pACYC184 derivative expressing *Msm rpsO* under *E. coli rpsO* promoter and *Msm* 5’UTR	This paper
pS15*_Msm_*(v3)	pACYC184 derivative expressing *Msm rpsO* under *E. coli rpsO* promoter and 5’UTR -AUG	This paper
pAMYC	pACYC184 derivative carrying *oriM*	This paper
pAMS15*_Msm_*	pAMYC expressing *Msm rpsO* underthe *Msm rpsO* 5’-extended promoter (−231)	This paper
pAMS15*_Eco_*	pAMYC expressing *E. coli rpsO*	This paper
pMV261	replicative shuttle vector, Kan^r^	[22]
pMV306hsp	integrative shuttle vector bearing *hsp60* promoter and *rrnB* terminator, Kan^r^	[22]
pMVrpsO*_Msm_*:egfp	pMV306 derivatives bearing *egfp* fused with *Msm rpsO* 5’UTR under *rpsO* promoters differing in 5’ extensions (−47, −117 and −231 bp from TSS)	This paper
pMVrpsO*_Msm_*mut:egfp	pMV306 derivative bearing *egfp* fused with *Msm rpsO* 5’UTR harboring the mutated pseudoknot under the *rpsO* extended (−231) promoter	This paper
pMVrpsO*_Mtb_*:egfp	pMV306 derivative bearing *egfp* fused with *Mtb rpsO* 5’UTR under the *Mtb rpsO* promoter (−158)	This paper

*#* pQE30_egfp, a derivative of a standard vector pQE30 (Qiagen, Hilden, Germany) bearing the *egfp* gene cloned into MCS using BamHI and HindIII restriction sites, was provided by Prof. K.A. Lukyanov (Shemyakin-Ovchinnikov Institute of Bioorganic Chemistry RAS). The *egfp* gene encodes the enhanced green fluorescence protein (eGFP), an engineered mutant variant of the wild-type GFP, with brighter fluorescence.

## Data Availability

Not applicable.
